# Sugarcane microRNA shy-miR164 regulates sugar metabolism through direct cleavage of the transcription factor *ScNAC* mRNA

**DOI:** 10.1093/plphys/kiaf354

**Published:** 2025-08-07

**Authors:** Miao Wang, Ao-Mei Li, Fen Liao, Zhong-Liang Chen, Cui-Xian Qin, Bao-Qing Zhang, Xin-Yi Li, Ze-Lin Su, You-Qiang Pan, Dong-Liang Huang

**Affiliations:** Guangxi Key Laboratory of Sugarcane Genetic Improvement, Key Laboratory of Sugarcane Biotechnology and Genetic Improvement (Guangxi), Ministry of Agriculture and Rural Affairs, Sugarcane Research Institute, Guangxi Academy of Agricultural Sciences, Nanning 530007, China; Guangxi Key Laboratory of Sugarcane Genetic Improvement, Key Laboratory of Sugarcane Biotechnology and Genetic Improvement (Guangxi), Ministry of Agriculture and Rural Affairs, Sugarcane Research Institute, Guangxi Academy of Agricultural Sciences, Nanning 530007, China; Guangxi Key Laboratory of Sugarcane Genetic Improvement, Key Laboratory of Sugarcane Biotechnology and Genetic Improvement (Guangxi), Ministry of Agriculture and Rural Affairs, Sugarcane Research Institute, Guangxi Academy of Agricultural Sciences, Nanning 530007, China; Guangxi Key Laboratory of Sugarcane Genetic Improvement, Key Laboratory of Sugarcane Biotechnology and Genetic Improvement (Guangxi), Ministry of Agriculture and Rural Affairs, Sugarcane Research Institute, Guangxi Academy of Agricultural Sciences, Nanning 530007, China; Guangxi Key Laboratory of Sugarcane Genetic Improvement, Key Laboratory of Sugarcane Biotechnology and Genetic Improvement (Guangxi), Ministry of Agriculture and Rural Affairs, Sugarcane Research Institute, Guangxi Academy of Agricultural Sciences, Nanning 530007, China; Guangxi Key Laboratory of Sugarcane Genetic Improvement, Key Laboratory of Sugarcane Biotechnology and Genetic Improvement (Guangxi), Ministry of Agriculture and Rural Affairs, Sugarcane Research Institute, Guangxi Academy of Agricultural Sciences, Nanning 530007, China; Guangxi Key Laboratory of Sugarcane Genetic Improvement, Key Laboratory of Sugarcane Biotechnology and Genetic Improvement (Guangxi), Ministry of Agriculture and Rural Affairs, Sugarcane Research Institute, Guangxi Academy of Agricultural Sciences, Nanning 530007, China; Guangxi Key Laboratory of Sugarcane Genetic Improvement, Key Laboratory of Sugarcane Biotechnology and Genetic Improvement (Guangxi), Ministry of Agriculture and Rural Affairs, Sugarcane Research Institute, Guangxi Academy of Agricultural Sciences, Nanning 530007, China; Guangxi Key Laboratory of Sugarcane Genetic Improvement, Key Laboratory of Sugarcane Biotechnology and Genetic Improvement (Guangxi), Ministry of Agriculture and Rural Affairs, Sugarcane Research Institute, Guangxi Academy of Agricultural Sciences, Nanning 530007, China; Guangxi Key Laboratory of Sugarcane Genetic Improvement, Key Laboratory of Sugarcane Biotechnology and Genetic Improvement (Guangxi), Ministry of Agriculture and Rural Affairs, Sugarcane Research Institute, Guangxi Academy of Agricultural Sciences, Nanning 530007, China

## Abstract

High sugar content is the primary objective in sugarcane (*Saccharum* spp. hybrids) breeding. Plant microRNA 164 (miR164) plays pivotal roles in plant development and stress responses through the post-transcriptional regulation of its target genes. NAC transcription factors play a crucial role in various plant physiological processes. However, their role in sugar metabolism remains uncharacterized. Our previous work revealed the potential role of the shy-miR164 and a sugarcane NAC transcription factor (ScNAC) in sugar metabolism based on multiomics analysis. In this study, shy-miR164 and ScNAC exhibited an inverse regulatory relationship in sugarcane. Subsequent RLM-RACE and dual-luciferase assays confirmed that shy-miR164 regulates ScNAC expression through direct cleavage of its mRNA. Knockdown of miR164 using short tandem target mimic technology significantly enhanced sugar content in tomato (*Solanum lycopersicum* L.) fruits, whereas the opposite effect was observed in miR164-overexpressing plants, indicating its involvement in sugar metabolism. Furthermore, heterologous expression of ScNAC in tomato also enhanced fruit sugar content. Taken together, these findings reveal a previously unexplored role of shy-miR164 in sugar metabolism through the direct cleavage of its target gene ScNAC. This work advances our understanding of the mechanisms underlying sugar metabolism and provides candidate targets for improved sugar production in sugarcane through biotechnology.

## Teaser

A sugarcane microRNA (mRNA) regulates sugar metabolism through cleaving the mRNA of an NAC transcription factor, providing mechanistic insights and potential molecular targets for improving sugar content.

## Introduction

Sugarcane (*Saccharum* spp. hybrids) is the most important sugar crop in the world, accounting for approximately 80% of global sugar production (Devarumath et al. 2019), and it is also the world's second largest bioenergy crop, with 40% of the world’s ethanol produced from sugarcane (Hillman et al. 2011; Vaz 2019). High sugar content has always been the primary goal in sugarcane breeding programs. As one of the most genetically complex species among cultivated crops, sugarcane is an aneuploid allopolyploid crop with a complicated genetic background and considerable segregation in hybrid offspring. These complexities render sugarcane conventional breeding highly labor-intensive, lengthy, and inefficient (Dunckelman and Legendre 1982). Consequently, sugar improvement through conventional breeding has progressed at a slow pace. Molecular breeding represents a promising strategy for enhancing sugar content in sugarcane. Elucidating the molecular mechanisms and identifying the key factors regulating sugar metabolism in sugarcane are essential for developing high-sugar varieties through molecular techniques.

MicroRNA (miRNA) is a class of endogenous non-coding small RNAs, 21 to 24 nt in length, and is broadly present in eukaryotes (Sun et al. 2019). miRNA is involved in regulating a wide range of processes, such as plant growth, cell differentiation, cell cycle, signal transduction, nutrient metabolism, and responses to biotic and abiotic stresses (Liu et al. 2010; Debat and Ducasse 2014; Nogoy et al. 2018; Liu et al. 2018a). miRNA regulates gene expression through base pairing with target genes' mRNAs (Jinek and Doudna 2009). Perfect or nearly perfect base pairing often leads to cleavage of target mRNAs, while incomplete base pairing mainly results in translational repression or degradation (Hutvágner and Zamore 2002; Meister et al. 2004; Song et al. 2004; Lim et al. 2005).

As a member of the highly conserved miRNA family in plants, microRNA 164 (miR164) primarily targets NAC (NAM, ATAF1/2, and CUC2) family transcription factors (Liu et al. 2022). NAC transcription factors are a class of plant-specific transcription factors with a highly conserved NAC domain at the N-terminus and a variable transcriptional regulatory region at the C-terminus (Ooka et al. 2003). miR164 plays key roles in plant growth, development, and responses to biotic and abiotic stresses by modulating its target gene NAC transcription factors (Wang et al. 2020; Zhan et al. 2021; Wang et al. 2021a). Overexpression of sly-miR164a in tomato (*Solanum lycopersicum* L.) decreased expression of NAC transcription factors and delays both pre- and postharvest fruit ripening (Wang et al. 2023). Furthermore, knockdown of sly-miR164a enhanced the postharvest chilling tolerance of tomato fruit during low-temperature storage (Zhao et al. 2022). miR164a-NAM3 module conferred cold tolerance in tomato plants via direct regulating the expression of 1-aminocyclopropane-1-carboxylate synthase 1A (*SlACS1A*), 1-aminocyclopropane-1-carboxylate synthase 1B (*SlACS1B)*, 1-aminocyclopropane-1-carboxylate oxidase 1 (*SlACO1*), and 1-aminocyclopropane-1-carboxylate oxidase 4 (*SlACO4*) (Dong et al. 2022). Moreover, miR164 also regulates the development of leaves and floral organs, organ fusion, shoot, and ﬂower boundary speciﬁcation, as well as drought resistance by modulating its target gene NAC transcription factors (Mallory et al. 2004; Zheng et al. 2019; Gupta et al. 2021).

In our previous work, we conducted a multiomic analysis on high-sugar and low-sugar sugarcane genotypes at 3 sugar accumulation stages, and preliminarily revealing the potential regulatory role of miRNA in sugar accumulation in sugarcane (Wang et al. 2022). Specifically, we found that an miR164 (shy-miR164) from *Saccharum* spp. hybrids and an NAC transcription factor (*ScNAC*) exhibited contrasting expression between high and low sugar genotypes, as well as at different sugar accumulation stages. Moreover, *ScNAC* was predicted to be the target gene of shy-miR164 based on degradome analysis (Wang et al. 2022). Based on these findings, we hypothesize that shy-miR164 may be involved in sugar metabolism in sugarcane by regulating the target gene, *ScNAC*.

To elucidate the role and underlying mechanisms of the shy-miR164-*ScNAC* module in sugar metabolism in sugarcane, we isolated shy-miR164 and *ScNAC* from sugarcane in the present study. Knockdown and overexpression of homologous shy-miR164 in tomato indicated that shy-miR164 regulated sugar metabolism. Subsequent RLM- RACE and dual luciferase assay confirmed that shy-miR164 directly cleaved *ScNAC* mRNA. Moreover, overexpression of *ScNAC* in tomato demonstrated that *ScNAC* also participates in sugar metabolism. This work demonstrates that miR164 regulates sugar metabolism by direct cleavage of its target gene, the NAC transcription factor.

## Results

### shy-miR164 and *ScNAC* exhibit a mutually inverse regulatory relationship in sugarcane

**Figure 1.**
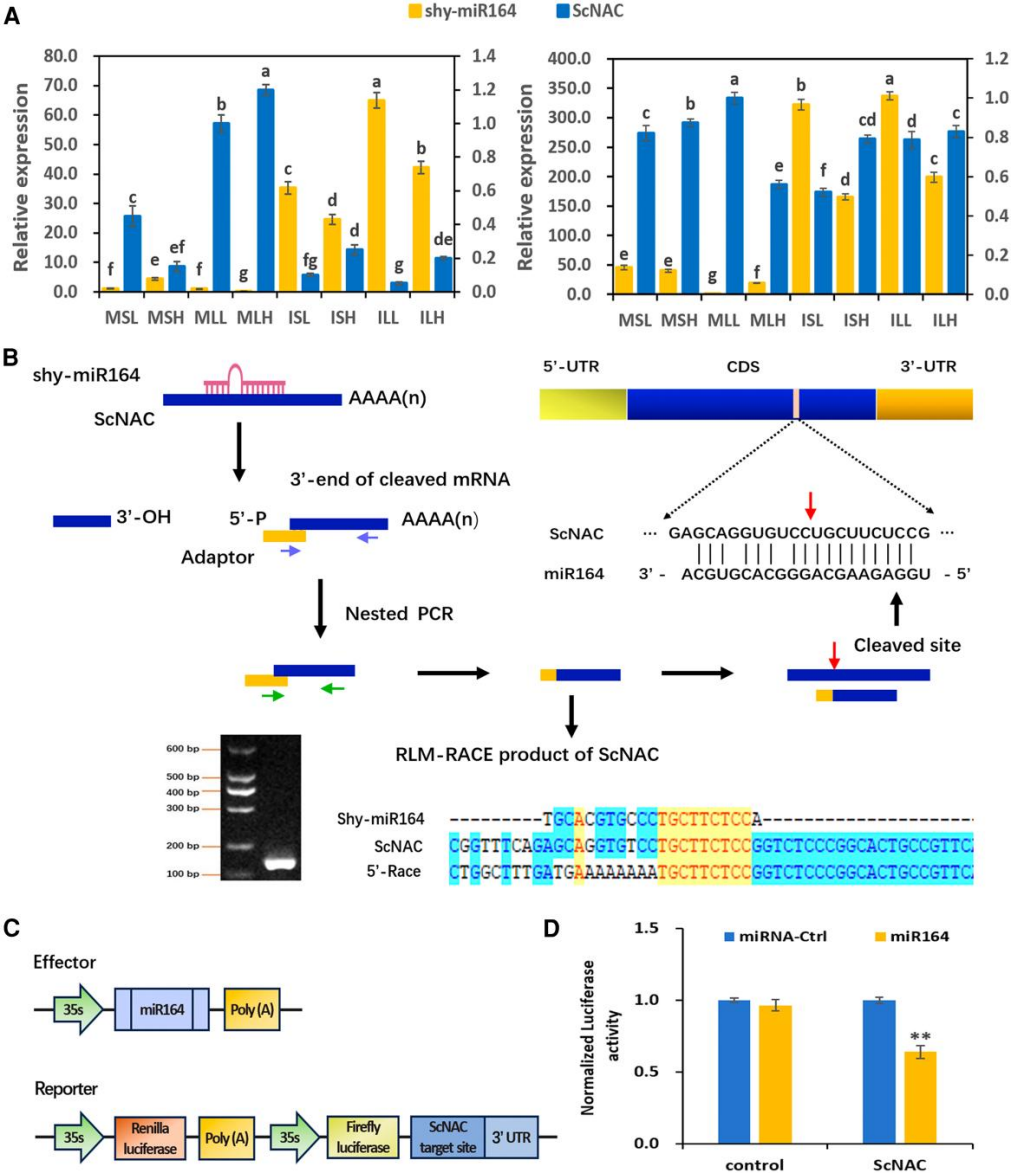
Shy-miR164 regulated the expression of its target gene *ScNAC* through direct cleavage of the mRNA. **A)** Expression profiles of shy-miR164 and its target gene, *ScNAC* in early (left) and late (right) stages of sucrose accumulation. Values shown are means ± SD (*n* = 3). Different letters indicate significant differences between samples (*P* < 0.05) as determined by a 1-way ANOVA followed by post hoc Tukey HSD analysis. MSL, mature stems of low-sugar genotype; MSH, mature stems of high-sugar genotype; MLL, mature leaves of low-sugar genotype; MLH, mature leaves of high-sugar genotype; ISL, immature stems of low-sugar genotype; ISH, immature stems of high-sugar genotype; ILL, immature leaves of low-sugar genotype; ILH, immature leaves of high-sugar genotype. **B)** Identification of the shy-miR164 cleavage site on the target mRNA by RLM-RACE. The red arrow above the *ScNAC* sequences indicates the cleavage site located between the 10th and 11th nucleotides of shy-miR164. The yellow-highlighted sequence represents the consensus region among the shy-miR164, ScNAC, and RACE-derived sequences, indicating the miRNA-mediated cleavage site on ScNAC mRNA. The purple and green arrows represent the outer and inner primers of nested PCR. CDS, coding sequence; UTR, untranslated region; RLM-RACE, RNA ligase-mediated rapid amplification of cDNA ends. **C)** Schematic representation of the dual-luciferase assay used to assess the regulation of *ScNAC* by shy-miR164. **D)** Dual-luciferase assay results showing the suppression of luciferase activity by shy-miR164. The bars (from left to right) represent the following vector combinations: LUC + SK, LUC + SK-shy-miR164, LUC-ScNAC + SK, and LUC-ScNAC + SK-shy-miR164. The luciferase activity was quantified as the normalized ratio of firefly luciferase to Renilla luciferase (LUC/REN). Values shown are means ± SD (*n* = 3). Asterisk indicates significant differences between treatments and the control (**P < 0.01, Student's *t*-test).

In our previous study, a multiomic analysis identified an inverse expression profile of the miR164-*NAC* (*ScNAC*) pair in sugarcane with varying sucrose content at different stages of sucrose accumulation, suggesting their potential mutual involvement in sucrose accumulation (Wang et al. 2022). To validate the expression patterns of shy-miR164 and its potential target gene, *ScNAC*, we conducted stem-loop RT-qPCR and RT-qPCR analyses on immature and mature stems and leaves from low- and high-sugar sugarcane genotypes at both early and late stages of sucrose accumulation. shy-miR164 exhibited higher accumulation in immature tissues compared to mature tissues. Furthermore, significant differences in shy-miR164 levels were observed between the high-sugar and low-sugar genotypes at both stages of sucrose accumulation. Notably, the expression of shy-miR164 was inversely associated with the expression of its target gene, *ScNAC*, across all tissues and stages, thereby confirming the inverse regulatory relationship between shy-miR164 and *ScNAC* (Fig. 1A).

### shy-miR164 directly targeted *ScNAC* through cleavage

To confirm whether *ScNAC* is a direct target of shy-miR164, we performed modified 5′ RNA ligase-mediated rapid amplification of cDNA ends (RLM-RACE). After reverse transcription, nested PCR amplification was conducted using gene-specific primers and adapter primers, yielding a target band of the expected size (Fig. 1B). Sequencing of the fragment revealed that the cleavage site was located between the 10th and 11th nucleotides of shy-miR164 (Fig. 1B). Additionally, dual-luciferase reporter assays were employed to investigate the in vivo regulatory interaction between shy-miR164 and *ScNAC* (Fig. 1C). When shy-miR164 was co-expressed with a luciferase construct containing *ScNAC* target sites, luciferase activity (LUC/REN ratio) was significantly reduced, indicating that shy-miR164 suppressed *ScNAC* expression through its target sequences (Fig. 1D). These results collectively confirm that shy-miR164 directly regulates *ScNAC* through cleavage in sugarcane.

### shy-miR164 mature sequence is highly conserved in plants

**Figure 2.**
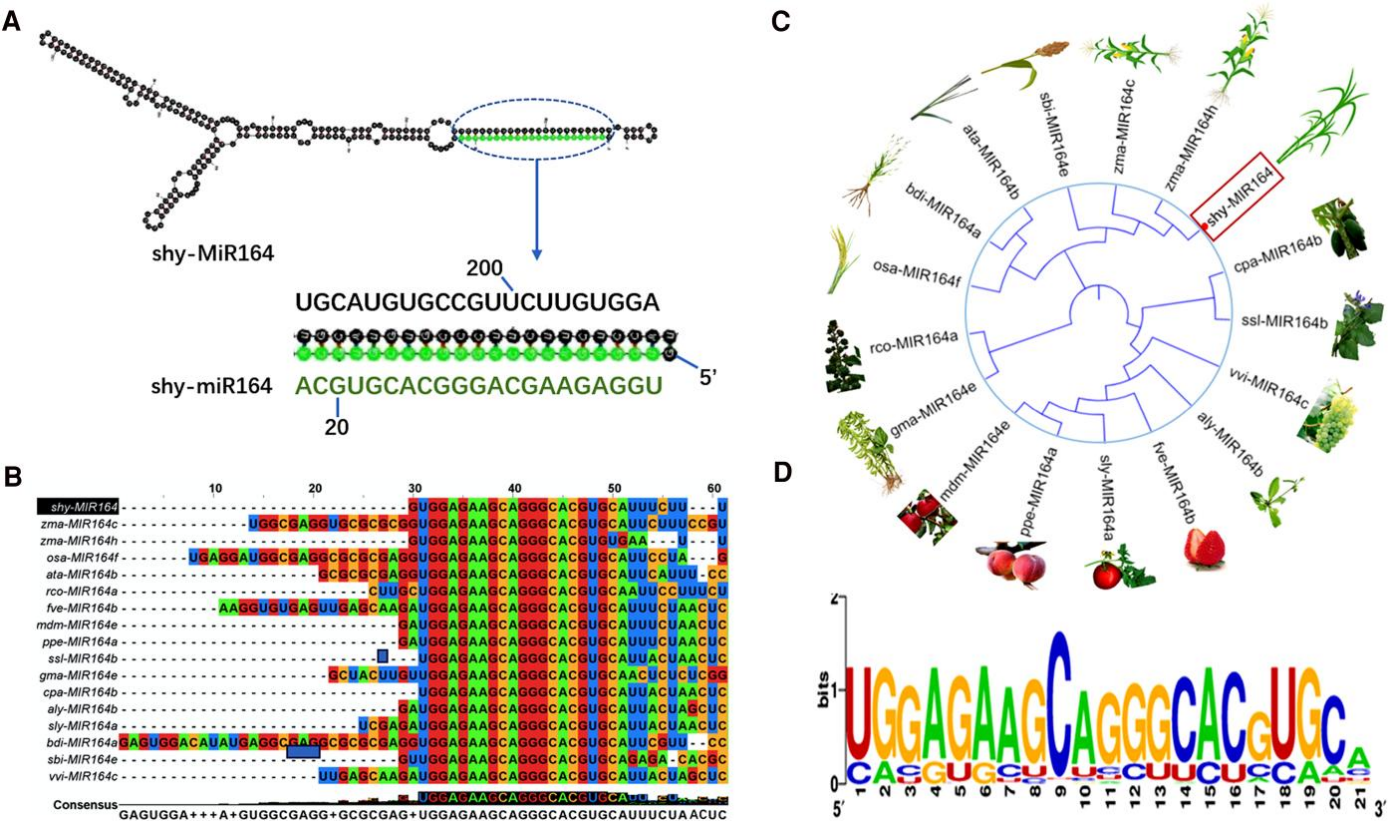
Analysis of miR164 sequences. **A)** Predicted hairpin structure of the sugarcane miR164 precursor. The mature miR164 sequence is highlighted in green. **B)** Sequence alignment of miR164 precursors from 16 plant species, showing the regions of the mature miR164 sequences. Different colors represent distinct nucleotide bases. Aly, *Arabidopsis lyrate*; Ata, *Aegilops tauschii*; Bdi, *Brachypodium distachyon*; Cpa, *Carica papaya*; Fve, *Fragaria vesca*; Gma, *Glycine max*; Mdm, *Malus domestica*; Osa, *Oryza sativa*; Ppe, *Prunus persica*; Rco, *Ricinus communis*; Sbi, *S. bicolor*; Sly, *S. lycopersicum*; Ssl, *Salvia sclarea*; Shy, *Saccharum* spp. hybrids; Vvi, *Vitis vinifera*; Zma, *Z. mays*. **C)** Phylogenetic analysis of miR164 precursor sequences from 16 plant species, showing the evolutionary relationship among the miR164 precursors. **D)** Sequence logo view of the mature miR164 sequence based on 165 mature miR164 sequences obtained from the miRBase database, highlighting conserved regions. The total stack height shows sequence conservation at that position, while the height of each letter represents the relative frequency of the nucleic acid.

Since the sugarcane miR164 has not been previously reported in the miRBase database, we cloned the precursor sequence of shy-miR164 along with its flanking regions, based on miRNA sequencing data from our previous study (Wang et al. 2022). The shy-miR164 precursor is 224 nucleotides in length and was predicted to form a typical stem-loop structure, with a minimal folding free energy of −118.50 kcal/mol. The mature 21 nt shy-miR164 sequence was found at the 5′ end of the precursor (Fig. 2A). To further investigate the evolutionary conservation of shy-miR164, we aligned the sugarcane precursor sequence with the miR164 precursor sequences from 15 plant species available in the miRBase database (Fig. 2B). Phylogenetic analysis revealed that the sugarcane miR164 precursor, shy-miR164, clustered with maize miR164 (zma-MIR164 h) in a single branch, sharing 83.25% identity (Fig. 2C). Despite sequence variations in miR164 precursors across different species, the first 20 nucleotides of the mature miR164 sequence were highly conserved between monocot and dicot plants (Fig. 2D).

### miR164 regulates growth and maturation as well as sugar metabolism

**Figure 3.**
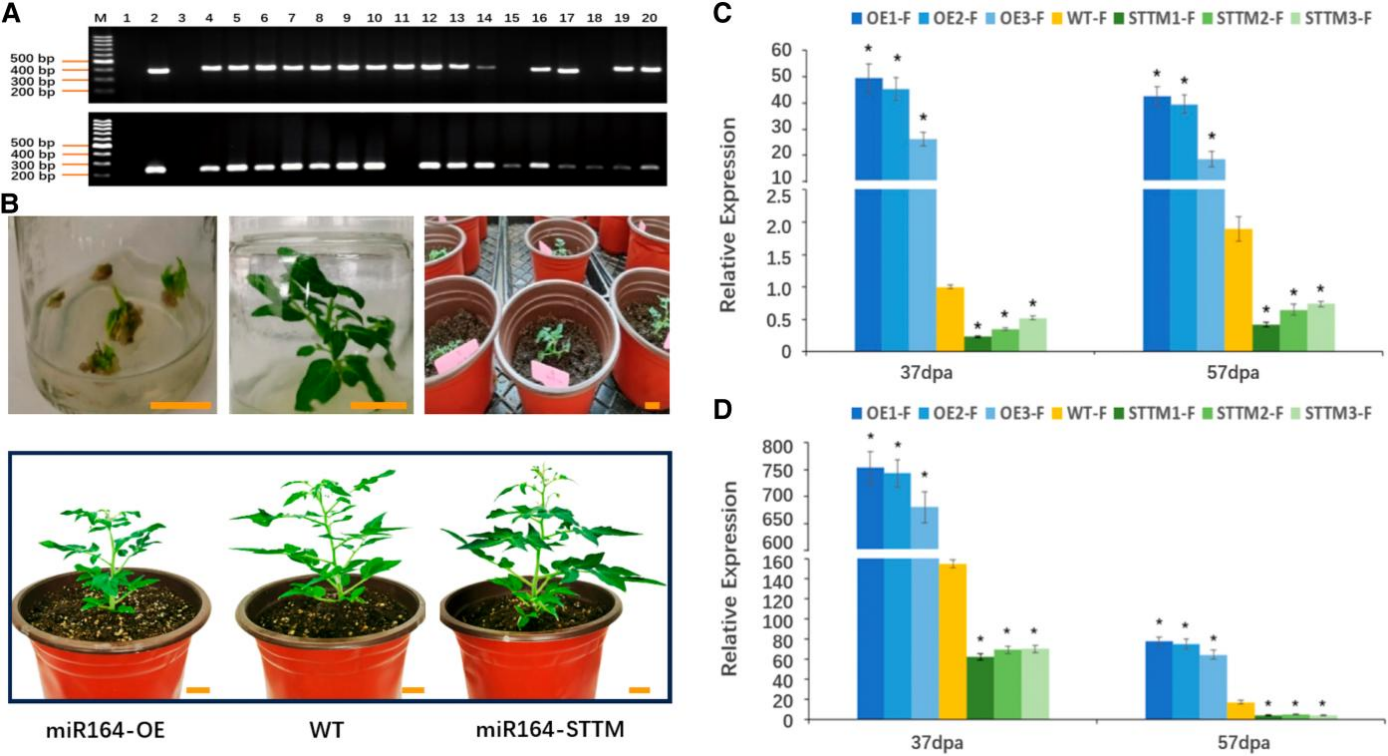
Generation of transgenic plants. **A)** Identification of transgenic lines by PCR. M, DNA ladder; 1, control without DNA template; 2, control plasmid; 3, WT; 4 to 20, PCR detection of genetically modified (GM) lines following selection medium screening. **B)** Selection and transplantation of tomato transgenic lines. Scale bar: 2 cm. Images were digitally extracted for comparison. **C)** and **D)** Abundance of miR164 in leaves (C) and fruits (D) of miR164-OE, miR164-STTM, and WT tomatoes. Values shown are means ± SD (*n* = 3). Asterisks denote statistically significant differences relative to the WT (*P < 0.05, Student's *t*-test).

To investigate the functions of shy-miR164, we overexpressed and silenced miR164 in tomato plants, utilizing the conserved, homologous sequence of shy-miR164 and tomato miR164. We obtained 15 independent lines of miR164-silenced plants and 16 independent lines of miR164-overexpressing plants (Fig. 3A and B). To assess the expression levels of miR164 in the transgenic lines, leaves and fruits were collected from the plants at 37 days post-anthesis (dpa) and 57 dpa for stem-loop RT-qPCR analysis. Compared to the wild-type (WT), miR164 abundance was significantly reduced in the silenced lines, indicating that the short tandem target mimic (STTM) vector effectively silenced miR164 in the tomato plants. In the overexpressing lines, miR164 abundance was significantly elevated, ranging from 5 to 49 times the control level across different tissues (Fig. 3C and D). Although shy-miR164 expression was detected in all samples, it was predominantly expressed in the fruits. Furthermore, miR164 abundance was significantly elevated in the fruits at 37 dpa and subsequently decreased at 57 dpa. The specific accumulation of miR164 in the fruits during the ripening stages suggests its critical role in fruit ripening.

**Figure 4.**
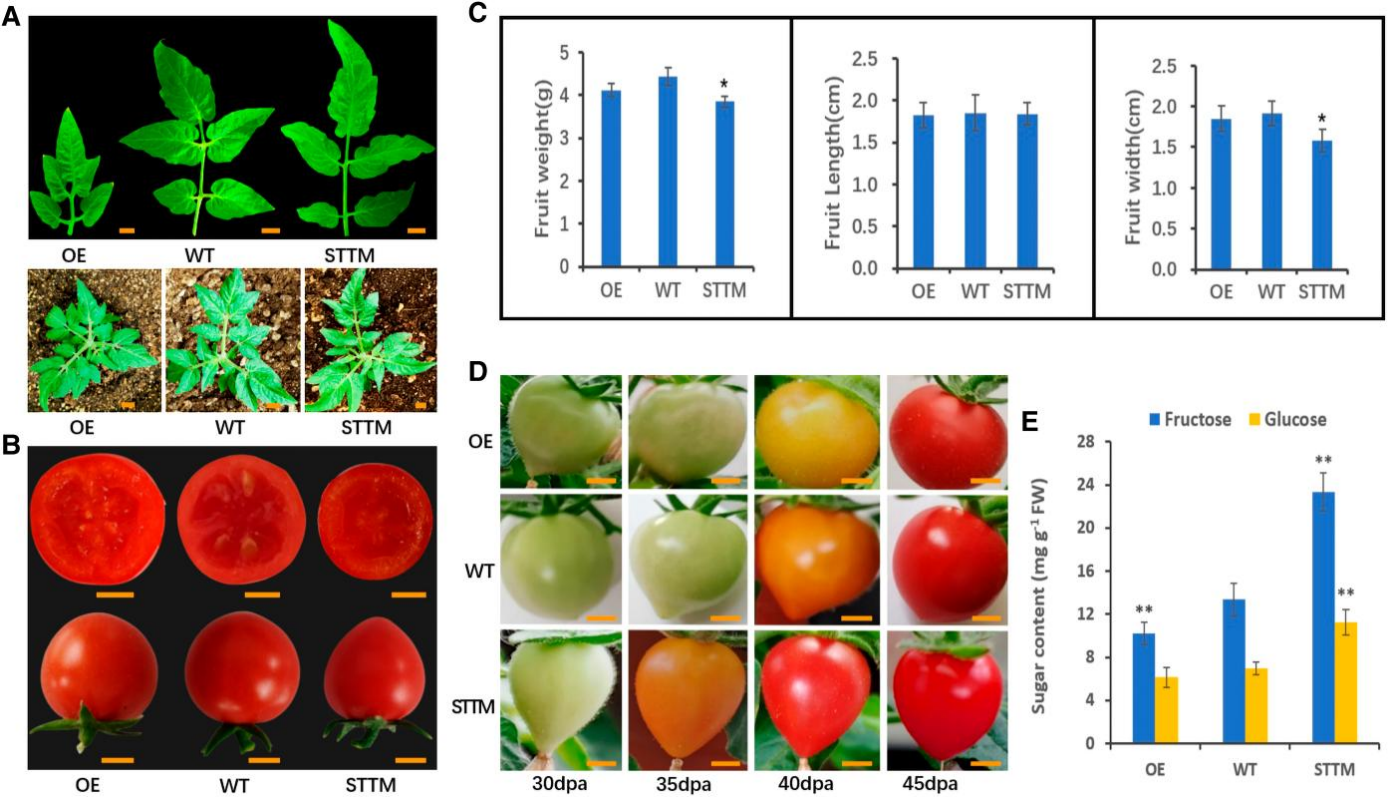
Phenotypes and sugar content of miR164-OE and miR164-STTM tomato plants. **A)** Phenotypic differences in leaves. Images were digitally extracted for comparison. Scale bar: 1 cm. **B)** and **C)** Phenotypic differences in fruits. Scale bar: 0.5 cm. Images were digitally extracted for comparison. Bar graphs show the means ± SD (*n* = 5). Asterisks denote significant differences between transgenic lines and the WT (*P < 0.05, Student's *t*-test). **D)** Fruit ripening phenotypes of miR164 transgenic lines and WT at 30, 35, 40, and 45 dpa. Scale bar: 0.5 cm. **E)** Fructose and glucose contents of miR164-OE and miR164-STTM fruits. Values shown are means ± SD (*n* = 3). Asterisks indicate significant differences between transgenic lines and the WT (**P < 0.01, Student's *t*-test).

Notably, the leaves of miR164 overexpression (miR164-OE) plants were smaller, with both leaf length and width substantially reduced compared to the WT and miR164-STTM plants. Additionally, compared to WT, miR164-OE plants failed to develop normal leaf margin serrations, while miR164-STTM plants exhibited deeper serrations (Fig. 4A). Although mature fruits of miR164-OE transgenic plants showed no marked differences in shape and color compared to the control, a notable discrepancy was observed in the number of locules, with WT fruits having 3 locules and overexpressing fruits having only 2 (Fig. 4B). The fruit length, fruit width, and single fruit weight of mature miR164-overexpressing plants did not show significant differences compared to the control, while STTM plant fruits were slightly smaller, had a larger fruit index, and contained fewer seeds (Fig. 4C).

At 35 dpa, when the miR164-STTM fruits started to turn orange, the fruits of miR164-OE and WT plants remained green. Furthermore, at 40 dpa, when the fruits of STTM plants were fully red, WT fruits began to turn orange, while the OE fruits were yellow. The fruits of STTM, OE, and WT plants all turned red at 45 dpa (Fig. 4D). The results indicated that the ripening of STTM-miR164a fruits was accelerated by 5 to 7 days compared to WT, while the ripening of miR164-OE fruits was delayed.

Sugar content analysis revealed that fructose and glucose were the main soluble sugars accumulated in tomato fruits, with fructose content approximately twice that of glucose. In the fruits of miR164-STTM, the glucose content was 11.25 mg/g, an increase of 61.17% compared to the WT fruits. The fructose content was 23.35 mg/g, 9.99 mg/g higher than the control, indicating a 74.78% increase. In contrast, the fructose content in the fruit of miR164-OE plants was 10.21 mg/g, and the glucose content was 6.14 mg/g, which were 23.58% and 12.03% lower than the controls, respectively (Fig. 4E). These results indicate that shy-miR164 plays a vital role in regulating sugar metabolism.

### ScNAC is an NAC transcription factor localized in the nucleus

**Figure 5.**
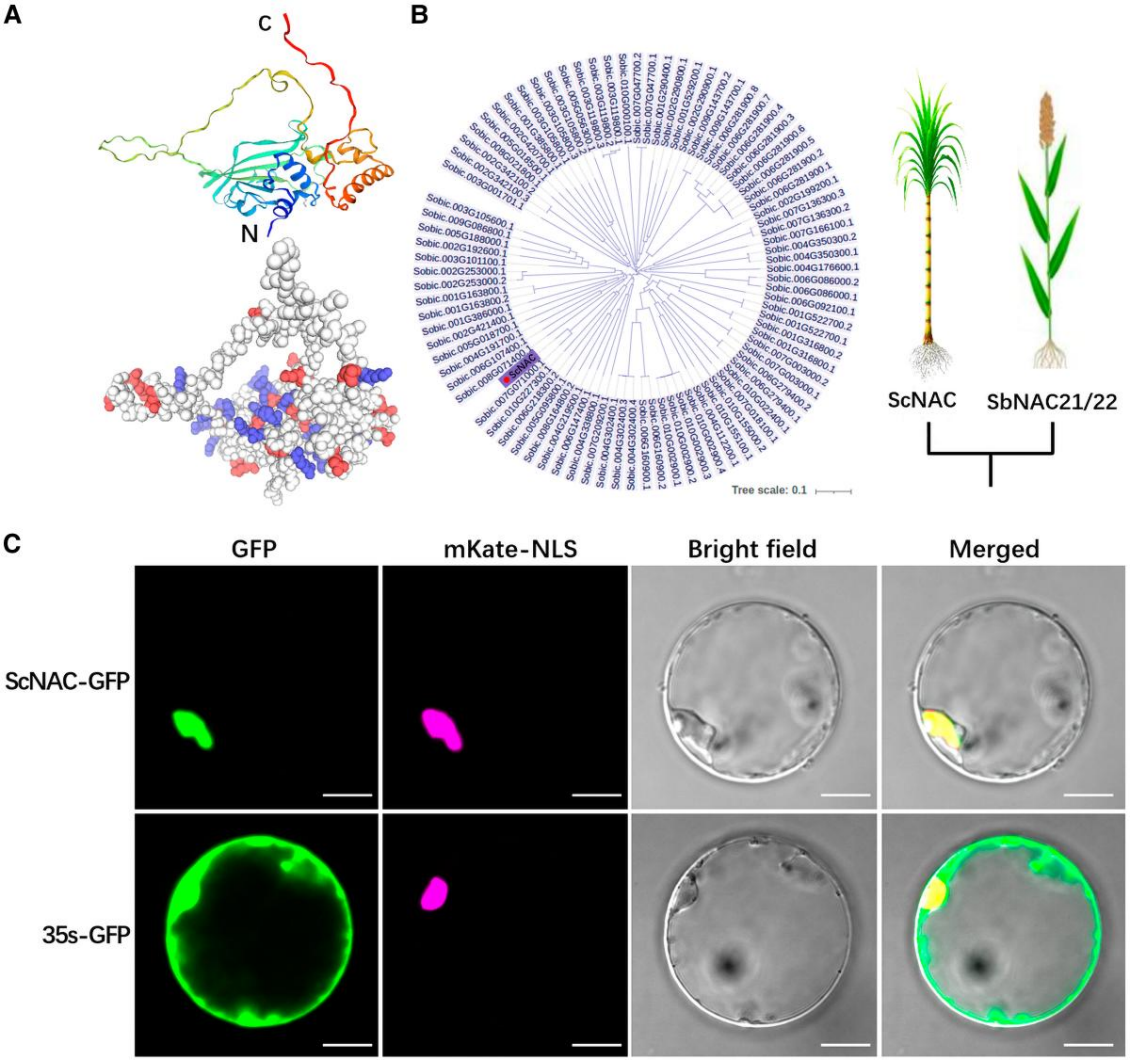
Analysis and subcellular localization of the ScNAC protein. **A)** Predicted tertiary structure of the ScNAC protein. The upper color scheme represents the sequence from N-terminus (N) to C-terminus (C), transitioning from blue to red. The lower color scheme uses red to represent negative charges, and blue to represent positive charges. **B)** Phylogenetic analysis of ScNAC and NAC in *S. bicolor*. Scale bar: 0.1 substitutions per site. **C)** Subcellular localization of ScNAC in rice protoplasts. ScNAC–GFP represents the fusion protein of ScNAC with GFP. 35s-GFP serves as the control for the empty vector, and the mKate-NLS construct was used as a nuclear control. Scale bar, 10 *μ*m. The mKate signals were pseudo-colored in magenta to ensure visibility for color vision deficiency, with non-pseudocolored images provided in Supplementary Fig. S1.

To elucidate how miR164 regulates sugar metabolism, further analysis was conducted on its target gene *ScNAC*, which was previously predicted through degradome sequencing. We isolated this gene from sugarcane (PQ663717). The open reading frame of the *ScNAC* gene is 858 bp in length, encoding 285 amino acids, with a theoretical molecular weight of 31.64 kDa and an isoelectric point of 8.48. The gene encodes an NAC protein that contains a highly conserved NAM domain (Pfam02365) at the N-terminus. The NAC protein comprises 48.77% random coils, along with extended chains (21.05%), α-helices (18.25%), and β-turns (11.93%) (Fig. 5A). A phylogenetic analysis was conducted by comparing the protein sequence with NAC transcription factors from sorghum (*Sorghum bicolor*), revealing that the protein clusters with NAC domain-containing protein 21/22 (Sobic.007G071000.1) from sorghum (Fig. 5B). Multiple sequence alignment of the ScNAC sequence with NAC domain-containing protein 21/22 sequences from other gramineous species, such as sorghum, maize (*Zea mays*), and wheat (*Triticum aestivum*), revealed highly conserved regions, particularly at the N-terminus. The protein sequence shares 90.53% and 84.70% similarity with the NAC domain-containing protein 21/22 sequences from sorghum and maize, respectively, suggesting ScNAC is an NAC21/22 member.

A GFP was fused to the C-terminal of ScNAC and co-transferred into rice protoplast with the mKate-NLS nuclear localization vector. The green fluorescence signal emitted by the target fusion protein overlapped with the red fluorescence signal from mKate-NLS, confirming the localization of the ScNAC protein to the cell nucleus (Fig. 5C).

### *ScNAC* participates in sugar metabolism

**Figure 6.**
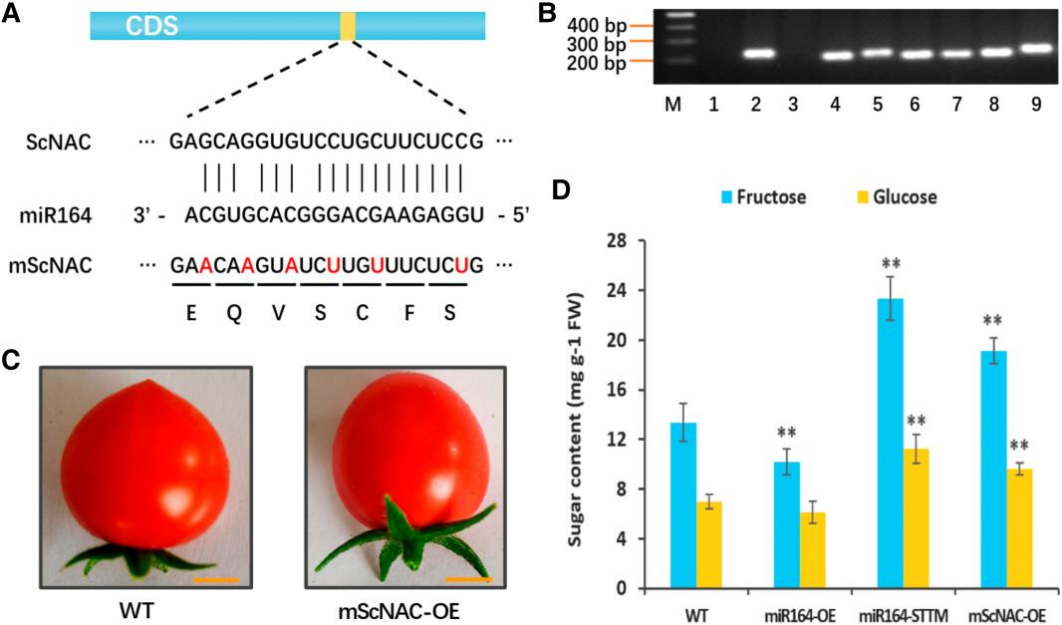
Sugar content of miR164-OE, miR164-STTM and m*ScNAC*-OE tomato fruits. **A)** Schematic diagram of miR164 target site mutation to generate the cleavage-resistant *ScNAC* (m*ScNAC*). The mutated sites are highlighted in red. **B)** Identification of m*ScNAC*-OE plants by PCR. Scale bar: 0.5 cm. M, DNA ladder; 1, control without DNA; 2, control plasmid; 3, WT; 4 to 9, PCR detection of GM lines. **C)** Fruits of m*ScNAC*-OE and WT tomatoes. **D)** Sugar contents (fructose + glucose) of miR164-OE, miR164-STTM, and m*ScNAC*-OE fruits. Values shown are means ± SD (*n* = 3). Asterisks indicate a statistically significant difference in the values (**P < 0.01, Student's *t*-test).

To clarify the involvement of *ScNAC* in sugar metabolism, we further generated transgenic tomatoes overexpressing cleavage-resistant *ScNAC* with mutations in the miR164 binding site that did not alter the amino acid sequences. Compared to wild plants, the fructose and glucose contents of the transgenic fruits were significantly increased (Fig. 6). The improvement in sugar content in m*ScNAC*-overexpressing plants was consistent with that in miR164-STTM plants. These results indicate that *ScNAC* also participate in sugar metabolism and mediated by shy-miR164.

## Discussion

miRNAs are a class of small non-coding RNAs derived from stem-loop precursors (Yu et al. 2017). The precursors of animal miRNAs typically range from 70 to 80 nt, whereas the length of plant miRNA precursors varies substantially, spanning tens to hundreds of nucleotides (Reinhart et al. 2002). The sugarcane shy-miR164 precursor identified in this study is 224 bp in length. Multiple sequence alignments revealed considerable variation in miR164 precursor sequences across different species. The sugarcane miR164 precursor sequence identified in this study exhibits high similarity to those in maize, with 2 highly similar precursors in maize measuring 216 and 270 bp, respectively. Most RNAs in organisms predominantly exist as single strands but can form secondary structures through base pairing and intermolecular hydrogen bonding. The formation of a stem-loop structure is a crucial step in miRNA maturation and a typical feature of miRNA precursors. The shy-miR164 precursor sequence identified in this study folds into a stable stem-loop structure with a minimum free energy of −118.50 kcal/mol. Free energy is a key indicator of RNA secondary structure stability, with lower values indicating greater stability (Sun et al. 2012). The low free energy predicted for the shy-miR164 precursor suggests its structural stability. miR164 is a conserved miRNA in plants (Chávez Montes et al. 2014). Despite extensive variation in miR164 precursor sequences, the mature sequences are highly conserved across both monocot and dicot plants. The shy-miR164 mature sequence identified in sugarcane is identical to the consensus sequence, suggesting the conservation of its function across different species.

As a highly conserved miRNA family in plants, the target genes of miR164 are also conserved. Current research indicates that the primary target of miR164 is the NAC family of transcription factors, which are plant-specific and play critical roles in various biological processes. Most miRNAs in plants regulate their target genes by binding to target sites, leading to the cleavage of target mRNAs, the RACE technique has been effectively applied to validate the interaction between miRNAs and their target genes (Sun et al. 2020). Using a modified RLM- RACE, we detected the 3′ end of *ScNAC* as a target cleaved by shy-miR164. The cleavage site was subsequently determined by aligning the fragment sequence with the mRNA and miRNA sequences. The dual-luciferase assay further confirmed the negative regulatory role of shy-miR164 on *ScNAC* through its target sites. These findings ensure that shy-miR164 regulates its target gene, *ScNAC* through direct cleavage.

miR164 is a crucial regulator in plants, involved in various significant biological processes (Chang et al. 2021; Zhan et al. 2021; Lin et al. 2022). However, its regulatory role in sugar metabolism has not been reported until now. To explore the regulatory role of miR164 in sugar metabolism, we generated transgenic tomatoes with both gain- and loss-of-function mutations in miR164 and observed that the abundance of miR164 significantly influenced the fructose and glucose content in tomato fruits. The sugar content in miR164-OE fruits decreased, while miR164 knockdown significantly increased the sugar content in tomato fruits. This report addresses the involvement of miR164 in sugar metabolism.

NAC transcription factors are widely distributed across plant species, from mosses to dicotyledonous plants. They play a crucial role in various plant biological processes, including growth and development, stress responses, phytohormone signaling, and the biosynthesis of secondary metabolites (Yamaguchi et al. 2010; Liu et al. 2018b; Mao et al. 2020; Martin-Pizarro et al. 2021; Zhan et al. 2021; Wang et al. 2021a, 2021b). Previous studies have highlighted the involvement of NAC21/22 members in stress responses. Specifically, *MhNAC21/22* has been shown to modulate resistance to *Alternaria alternata* in *Malus hupehensis* through the regulation of jasmonic acid signaling (Zhou et al. 2023). Similarly, *TaNAC21/22* plays a role in mediating wheat resistance to *Puccinia striiformis* (stripe rust) (Feng et al. 2014). Despite these findings, the specific role of NAC21/22 in sugar metabolism remains unknown. To clarify the involvement of the NAC21/22 member, *ScNAC* in sugar metabolism, we overexpressed *ScNAC* with mutations in the miR164-binding site in tomatoes. We found that elevated *ScNAC* levels enhanced sugar accumulation, consistent with the results of miR164 knockdown. This establishes that NAC21/22 member is involved in sugar metabolism. Taken together, these findings demonstrate that miR164 regulates sugar accumulation through its target gene *ScNAC*, providing important insights into the mechanism by which miR164 governs sugar accumulation in sugarcane.

The abundance of miR164 in fruits is substantially higher than in other tissues, and its levels increase notably during fruit ripening in plants (Liu et al. 2014 ; Rosas-Cárdenas et al. 2015). Similarly, we found that miR164 was detectable in both leaves and fruits of tomato, but its accumulation was particularly high in the fruit, especially during the ripening process. Further investigation revealed that miR164 plays a role in regulating tomato fruit ripening. Overexpression of miR164 delayed fruit ripening, resulting in firmer fruits, while knockdown of miR164 accelerated ripening. Specifically, overexpression of miR164a reduced the expression of tomato NAC transcription factors *NAM3*, *GOB*, and *NAC1*, inhibited ethylene biosynthesis, and decreased lycopene accumulation, thereby delaying fruit ripening (Wang et al. 2023). Ethylene is a critical regulator of plant development, particularly in fruit ripening. During tomato fruit ripening, ethylene levels continuously rise, peaking at the time of fruit maturity (Gupta et al. 2021). 1-aminocyclopropane-1-carboxylate synthase (ACS) and 1-aminocyclopropane-1-carboxylate oxidase (ACO) are key enzymes involved in the ethylene biosynthesis pathway in plants (Pattyn et al. 2021). The target gene of miR164, the NAC family transcription factor SlNAM3, can directly bind to the promoters of *SlACS1A*, *SlACS1B*, *SlACO1*, and *SlACO4*, activating their transcription and promoting ethylene biosynthesis. Knockdown of miR164 or overexpression of *SlNAM3* both led to increased ethylene levels in tomatoes, suggesting that miR164 regulates ethylene biosynthesis through its target NAC transcription factors (Dong et al. 2022).

**Figure 7.**
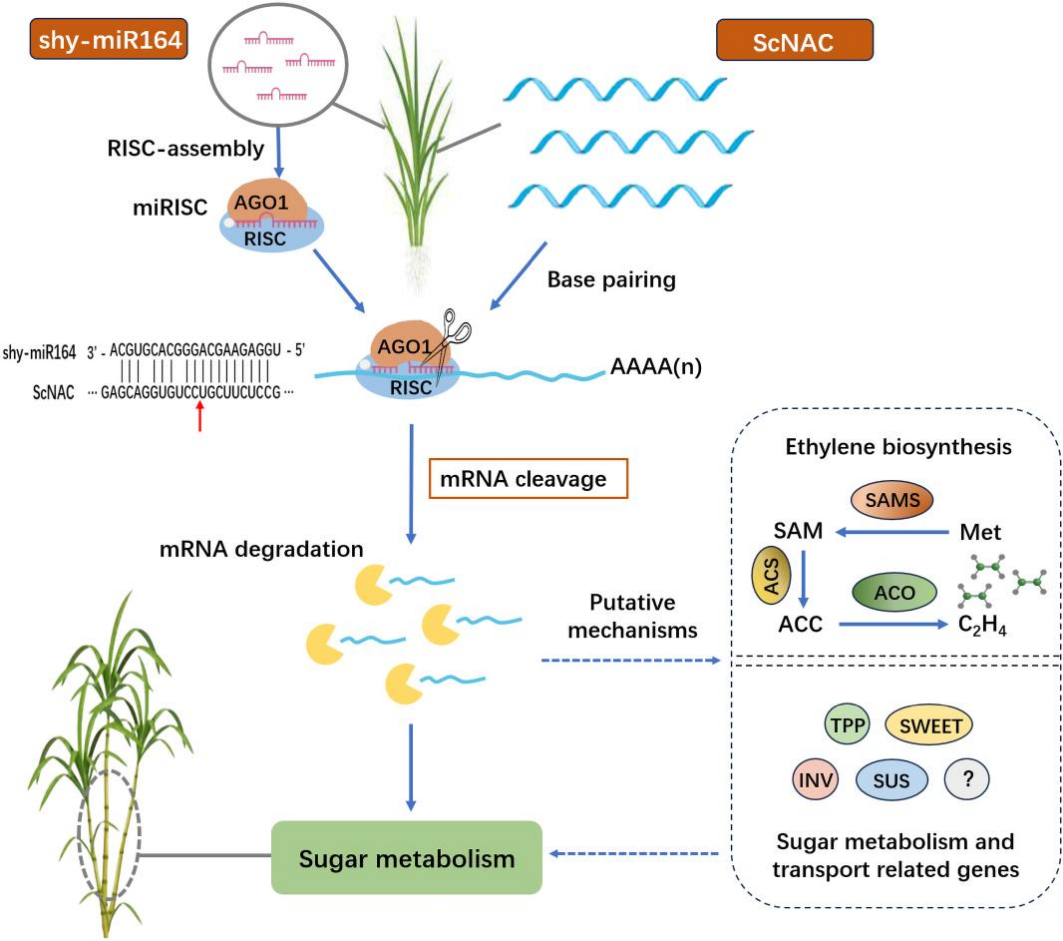
Schematic model illustrating miR164's regulation of sugar metabolism through the direct cleavage of *ScNAC*. In sugarcane, miR164 associates with Argonaute (AGO) proteins to form the miRNA-induced silencing complex (miRISC), which specifically binds to the coding region of *ScNAC* through sequence complementarity and mediates target mRNA cleavage, thereby leading to post-transcriptional downregulation of *ScNAC*. *ScNAC* modulates sugar metabolism through 2 potential mechanisms, including regulation of ethylene biosynthesis and control of sugar metabolism/transport-related proteins. The arrow beneath the *ScNAC* sequence indicates the shy-miR164 cleavage site on *ScNAC* mRNA. miRISC, miRNA-induced silencing complex; Ago1, Argonaute 1; Met, methionine; SAMS, *S*-adenosyl-L-methionine synthetase; SAM, *S*-adenosyl-L-methionine; ACS, 1-aminocyclopropane-l-carboxylate synthase; ACC, 1-aminocyclopropane-1-carboxylate; ACO, 1-aminocyclopropane-1-carboxylate oxidase; C_2_H_4_, ethylene; TPP, trehalose-6-phosphate phosphatase; SWEET, sugar will eventually be exported transporter; INV, invertase; SUS, sucrose synthase.

Additionally, ethylene induces the expression of miR164 precursor genes, thereby increasing miR164 levels (Lin et al. 2022), suggesting that the elevated abundance of miR164 during fruit ripening may be associated with ethylene induction. Ethylene also plays a critical role in the growth and sugar accumulation of sugarcane; the application of high ethephon concentrations during the sugar accumulation stage can accelerate maturation and enhance sugar content in sugarcane (Li et al. 2003; Solomon and Li 2004). Considering our findings and the role of miR164 and its target genes in ethylene biosynthesis, we propose that the miR164-NAC module could play a pivotal role in regulating sugar accumulation in sugarcane through its influence on ethylene biosynthesis (Fig. 7). Moreover, in many other crops, certain NAC transcription factors have been shown to regulate the expression of genes involved in sugar metabolism and transport, including trehalose-6-phosphate phosphatase 1 (*TPP1*), sugar will eventually be exported transporter 4 (*SWEET4*), sucrose synthase 2 (*SUS2*), and invertase (*INV*), by directly binding to their promoters (Li et al. 2021; Ren et al. 2021; Wang et al. 2021b; Li et al. 2022). This regulatory mechanism may also exist in sugarcane, potentially representing another pathway through which NAC transcription factors regulate sugar content. Nonetheless, the precise molecular interactions and signaling pathways that mediate this modulation in sugarcane remain to be thoroughly understood. Future research is required to uncover the complex mechanisms underlying the interplay among shy-miR164, ScNAC, ethylene signaling pathways, and sugar metabolism- and transport-related genes in sugar accumulation, which are essential for advancing our understanding of sugar metabolism and developing strategies to enhance sugar content in sugarcane.

## Conclusions

In this study, we isolated shy-miR164 and its target gene, *ScNAC*, in sugarcane and uncovered a pivotal role for shy-miR164 in sugar metabolism through the direct cleavage of *ScNAC*. These findings not only deepen our understanding of the regulatory mechanisms involved in sugar metabolism but also offer potential targets for enhancing sugar content in sugarcane through molecular breeding strategies. Further work should focus on enhancing sugar content in sugarcane by modulating the abundance of shy-miR164 or *ScNAC*.

## Materials and methods

### Plant materials

A high-sugar sugarcane (*Saccharum* spp. hybrids) genotype ROC22 (with average 15% sucrose content) and a low-sugar genotype GT 86 to 887 (with average 6% sucrose content) were planted in the experimental field of Sugarcane Research Institute, Guangxi Academy of Agricultural Sciences, Nanning, following the methods described in our previous work (Wang et al. 2022). Immature leaves and stems, and mature leaves and stems of 2 genotypes, were collected during the early and late stages of sugar accumulation for RT-qPCR experiments. Leaves of genotype ROC22 were used for gene cloning and RACE experiments. All samples were frozen in liquid nitrogen immediately and stored at −80 °C before use. The tomato variety Micro-Tom (*S. lycopersicum* L. cv. Micro-Tom) was cultivated in a light incubator at a temperature of 25 °C and a relative humidity of 80%. The photoperiod was set to a light/dark cycle of 16 h/8 h, with a photosynthetic photon flux density of 250 *μ*mol m^−2^ s^−1^.

### Cloning of sugarcane shy-miR164 precursors and *ScNAC*

For sugarcane miRNA shy-miR164 precursor cloning, genomic DNA from sugarcane leaves was extracted using the Plant Genome DNA Extraction Kit (Tiangen), following the manufacturer's instructions. Primers were designed based on the small-RNA library previously constructed by our laboratory (Wang et al. 2022) to amplify the miR164 precursor sequence and 200 bp flanking sequences. PCR amplification was performed with a program set as follows: 94 °C for 2 min, followed by 35 cycles of 95 °C for 20 s, 59 °C for 15 s, and 72 °C for 15 s. The products were gel purified, cloned, and sequenced.

For the shy-miR164 target gene NAC transcription factor (*ScNAC)*, total RNA was extracted from leaf samples using the Invitrogen Trizol reagent kit, and the first-strand cDNA was synthesized using the Fermentas RevertAid First Strand cDNA Synthesis Kit. The sequence of the target gene obtained from transcriptome sequencing was aligned with homologous genes in NCBI from related species such as sorghum (*S. bicolor*) and maize (*Z. mays*). Specific primers were designed using Primer 6.0 software. PCR was performed under the following conditions: sterile deionized water (15.00 *μ*L), 2 × PCR Buffer for KOD FX Neo (25.00 *μ*L), dNTPMix (10 mm) (1.00 *μ*L), KOD FX Neo (1 U/μL) (1.00 *μ*L), template (5.00 *μ*L), primer F (1.50 *μ*L), and primer R (1.50 *μ*L). PCR products were gel-purified and cloned before sequencing. The primers used in this work are listed in Supplementary Table S1.

### Sequence analysis of sugarcane shy-miR164 precursor and *ScNAC*

The hairpin structure of the shy-miR164 precursor was examined using the Mfold webserver. Precursor and mature sequences of miR164 from other species were obtained from miRbase (21.0), and multiple sequence alignments were performed using Clustal Omega. The conservation of miR164 mature sequences was analyzed using Weblogo (Crooks et al. 2004).

The basic physicochemical properties of the NAC protein were analyzed using the ProtParam tool (Wilkins et al. 1999). Protein conserved domain analysis was conducted via the NCBI Conserved Domain Database Search Service (https://www.ncbi.nlm.nih.gov/Structure/cdd/wrpsb.cgi), and the tertiary structure of the protein was predicted using Swiss-Model. Homology protein sequences of related species were retrieved from NCBI, sorghum NAC transcription factor sequences were obtained from the PlantTFDB database. Multiple sequence alignment was performed using Clustal Omega, and a phylogenetic tree was constructed using MEGA 11 with the Neighbor-Joining method and bootstrap analysis (1000 replicates).

### Subcellular localization of ScNAC

The coding sequence (CDS) of *ScNAC* was amplified and subsequently inserted into the pAN580 vector (linearized with restriction enzymes *Xba*I and *BamH*I) via homologous recombination. Primers used are listed in Supplementary Table S1. Enhanced green ﬂuorescent protein (eGFP) was fused to the C-terminal of ScNAC and driven by the *Cauliflower mosaic virus* (CaMV) 35S promoter. The plasmids were transferred into rice protoplasts with the nuclear localization vector mKate-NLS (the mKate signal can be detected in the nucleus). After 24 h of cultivation in the dark at 28 °C, eGFP, and mKate protein ﬂuorescence were detected using a confocal laser scanning microscope (Nikon C2-ER, Tokyo, Japan). The setup used for imaging was as follows: GFP laser, 488 nm; intensity, 4.0%; collection bandwidth, 500 to 540 nm; gain = 40; and mKate laser, 561 nm; intensity, 3.2%; and collection bandwidth, 580 to 640 nm; gain = 36.

### Reverse transcription quantitative PCR (RT-qPCR) analysis

Mature miR164 levels were quantified using stem-loop RT-qPCR. The stem-loop RT primer and qPCR primers for miR164 were designed according to Kramer (2011) and listed in Supplementary Table S1. Total RNA was extracted using RNAprep Pure Plant Kit and first-strand cDNA was synthesized using HiScript Ⅱ Q RT SuperMix for RT-qPCR according to the manufacturer's protocol. RT-qPCR was performed using the AnalytikJena-qTOWERE2.2 Real-Time PCR System (Germany) with SYBR Green (DBI, Roche). The reactions were performed as follows: 95 °C for 3 min, followed by 40 cycles of denaturation at 95 °C for 15 s, then annealing at 57 °C for 15 s and extension at 72 °C for 20 s. The sugarcane 18 s rRNA (Yang et al. 2016) and tomato Actin-7 (Candar-Cakir et al. 2016) were used as initial reference to normalize data.

The expression profiles of *ScNAC* were analyzed by RT-qPCR as previously described (Wang et al. 2022). The reactions were performed as follows: 95 °C for 3 min, followed by 35 cycles of denaturation at 95 °C for 10 s, then annealing at 57 °C for 10 s and extension at 72 °C for 20 s. The sugarcane glyceraldehyde-3-phosphate dehydrogenase (GAPDH/EF189713) gene was used as a reference gene. Primers used for amplifying are listed in Supplementary Table S1. All the reactions were performed with 3 replicates. The threshold cycle (CT) was determined and the 2^−ΔΔCt^ equation was used to calculate the mRNA and miRNA expression levels. Data were subjected to analysis of variance using SPSS18.0 (Analytical software, IBM, USA).

### RNA ligase-mediated rapid amplification of cDNA ends

To detect the shy-miR164 target site on *ScNAC* transcript, an improved RNA ligase-mediated rapid amplification of cDNA ends (RLM-RACE) was carried out using the First Choice RLM-RACE kit (Ambion Inc., Austin, TX, USA), omitting calf intestine alkaline phosphatase and tobacco acid pyrophosphatase treatment (Sun et al. 2020). Total RNA was obtained from sugarcane leaves and ligated to a 5′ RACE adapter. Based on the cloned mRNA sequence and the cleavage site identified through the degradome analysis, 2 gene-specific primers were designed to target the 3′ end of the cleavage site using Primer 6.0. The 5′ RACE outer primer and gene-specific outer primer were employed for the first round of nested PCR, followed by a second round using 5′ RACE inner primer and a gene-specific inner primer in accordance with the manufacturer's instructions. The PCR products were gel purified, cloned, and randomly selected for sequencing. The primer sequences are listed in Supplementary Table S1.

### Dual-luciferase assay

To conﬁrm that *ScNAC* expression was regulated by miR164, the dual-luciferase reporter system was employed. shy-miR164 sequence was inserted into pGreenII 62-SK vector via *Eco31*I restriction enzyme digestion and subsequent T4 DNA ligase-mediated connection, whereas the target site of miR164 in *ScNAC* was cloned into pGreenII 0800-LUC vector (linearized with restriction enzymes *Xba*I and *BamH*I) through homologous recombination. The primer sequences are listed in Supplementary Table S1. The vectors were co-transformed into *Arabidopsis* protoplast cells in different combinations (LUC + SK, LUC + SK-shy-miR164, LUC-ScNAC + SK, and LUC-ScNAC + SK-shy-miR164). After incubation at 23 °C for 20 h, fireﬂy luciferase (LUC) and Renilla luciferase (REN) activities were detected and quantified with a multimode reader (Victor Nivo, PerkinElmer) using the Dual Luciferase Reporter Assay System (Vazyme).

### Plasmid construction and tomato transformation

To overexpress shy-miR164, the miR164 mature sequence was inserted into the ath-miR319 precursor scaffold of the amiRNA expression vector derived from pCambia1300 (Ossowski et al. 2008; Yusuf et al. 2021). miR164 and the selection marker gene HPT were driven by the CaMV 35 s promoter, respectively. To block the function of miR164, the fragment consisting of 2 copies of miR164 linked by a 48 nt spacer was artificially synthesized followed by the short tandem target mimic (STTM) method of Yan (Yan et al. 2012). The miR164 STTM fragment was inserted downstream of the CaMV 35s promoter in the modified expression vector pCambia1300 to construct the silenced vector. The target fragments were inserted into the vectors by restriction enzyme *Eco31*I digestion and T4 DNA ligase-mediated ligation. For overexpression of *ScNAC*, the CDS of *ScNAC* with mutation in the binding site without change of amino acid sequence was synthesized. Then, the mutated *ScNAC* sequence was amplified and inserted downstream of the CaMV 35s promoter in the modified expression vector pCambia1300 (linearized with restriction enzyme *Eco31*I) via homologous recombination. The primer sequences are listed in Supplementary Table S1. The recombinant vectors were confirmed by sequencing, and then transformed into *Agrobacterium tumefaciens* strain GV3101. Genetic transformation of Micro-Tom tomato was carried out according to the method previously described (Qin et al. 2016).

### Phenotypic characterization of transgenic plants

Phenotypic data of overexpressing miR164 (miR164-OE) and silenced miR164 (miR164-STTM) plants were collected following the methods previously described (Li et al. 2005). At the mature stage of the third truss of fruit, the length and width of the largest leaves in the middle part of the plant were measured, and the leaf shape and lobation were observed. The fruit maturation period was recorded to assess the maturation time of different lines. The shape and the weight of the mature fruit, the number of locules, as well as longitudinal and transverse diameters of the fruit, were measured. For each treatment, 5 plants were selected and 3 leaves and fruits from each plant were measured, with WT plants as control.

### Sugar content analysis

Glucose, fructose, and sucrose contents were qualified in the fruits of miR164 overexpression (miR164-OE), miR164 silencing (miR164-STTM), *ScNAC* overexpression (*ScNAC*-OE), and WT tomatoes. In total, 9 fruits from 3 plants for each treatment were collected. After grinding into a homogenate, 5 g of sample was taken from each treatment, and gently mixed with zinc acetate solution and potassium ferrocyanide solution, making up to 25 mL. After 30 min of ultrasonic treatment, the samples were initially filtered through filter paper and 0.45 *μ*m aqueous phase filter membrane for use. The detection of sugar content was performed using high-performance liquid chromatography-evaporative light scattering detection on a liquid chromatograph (LC-20AT, Shimadzu) under the following conditions: a welchrom-XB-NH2 (4.6 mm × 250 mm, 5 *μ*m) column was used, with acetonitrile as mobile phase A and water as mobile phase B, at a volume ratio of 80:20 for A:B, a flow rate of 1 mL/min, a column temperature of 40 °C, and an injection volume of 20.00 *μ*L. The evaporative light scattering detector had a drift tube temperature of 85 °C and a nitrogen gas flow rate of 2 L/min. Glucose, fructose, and sucrose standards were weighed to prepare standard solutions. Linear regression equations and correlation coefficients were established based on the injection volume and peak area. The chromatographic peaks of sugars in the samples were identified according to the retention time of the standards, and the content of each type of sugar in the samples was quantitatively determined based on the peak area of the samples.

### Statistical analysis

Statistical analyses were conducted using SPSS 18.0 (IBM, USA). All experiments included at least 3 biological replicates. Data were analyzed by one-way ANOVA followed by post hoc Tukey HSD analysis or Student's t-test, with significance levels set at *P* < 0.05 or *P* < 0.01. Specific statistical methods for each result presented are detailed in the corresponding figure legends.

### Accession numbers

Sequence data from this article can be found in the NCBI/PlantTFDB/miRBase data libraries under accession numbers listed in Supplementary Table S2.

## Supplementary Material

kiaf354_Supplementary_Data

## Data Availability

The data underlying this article are available within the article.

## Supplementary data

The following materials are available in the online version of this article.

**Supplementary Table S1.** Primers used in this study.

**Supplementary Table S2.** MiRNAs and proteins in this study.

**Supplementary Figure S1.** Subcellular localization of ScNAC (native fluorescence of Fig. 5C).

## Dive Curated Terms

The following phenotypic, genotypic, and functional terms are of significance to the work described in this paper:

CUC2 Gramene: At5g53950
CUC2 Araport: At5g53950

sugar content Planteome: TO:0000333

ACS Gramene: AT5G36880
ACS Araport: AT5G36880

ethylene CHEBI: CHEBI:29362

GLUCOSE CHEBI: CHEBI:17234

NAC CHEBI: CHEBI:7421

1-AMINOCYCLOPROPANE-1-CARBOXYLATE CHEBI: CHEBI:30526

NAC1 Gramene: Solyc07g066330
NAC1 Araport: Solyc07g066330

NCBI Gramene: https://www.ncbi.nlm.nih.gov
NCBI Araport: https://www.ncbi.nlm.nih.gov
