# Supplementary material for: Sugarcane microRNA shy-miR164 regulates sugar metabolism through direct cleavage of the transcription factor ScNAC mRNA
Source: Plant Physiol. 2025 Aug 7;198(4):kiaf354. doi: 10.1093/plphys/kiaf354 (PMC12371838; doi:10.1093/plphys/kiaf354)
Supplement: kiaf354_Supplementary_Data [file kiaf354_supplementary_data.zip › PLPHYS-2025-0914R1_Supplementary Figure.pdf]

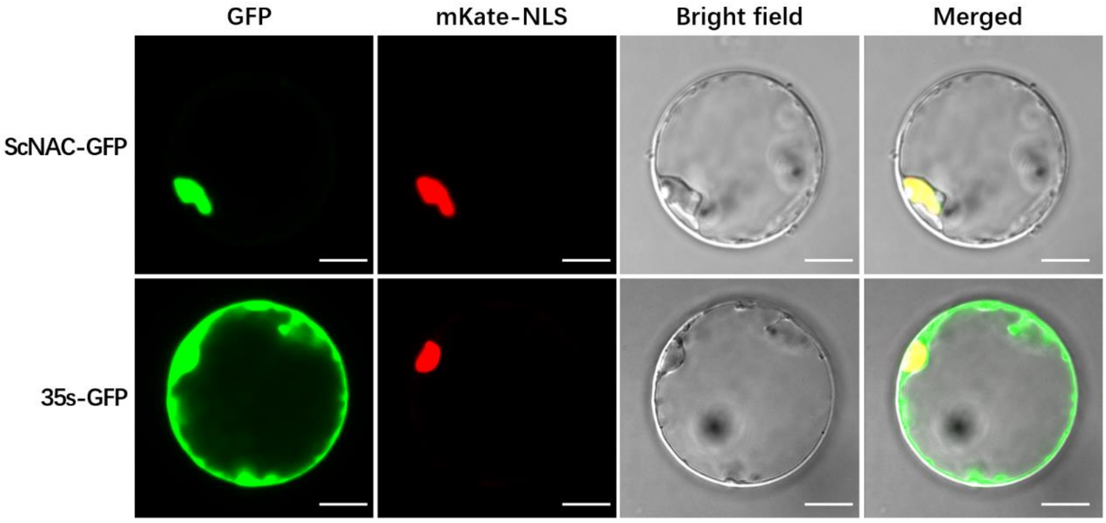

Supplementary Figure S1. Subcellular localization of ScNAC (native fluorescence of Fig 5C). ScNAC-GFP represents the fusion protein of ScNAC with GFP. 35s-GFP serves as the control for the empty vector, and the mKate-NLS construct was used as a nuclear control. Scale bar, 10 μm.
